# Elevated c-di-GMP Levels and Expression of the Type III Secretion System Promote Corneal Infection by Pseudomonas aeruginosa

**DOI:** 10.1128/iai.00061-22

**Published:** 2022-08-01

**Authors:** Joey Kuok Hoong Yam, Thet Tun Aung, Song Lin Chua, Yingying Cheng, Gurjeet Singh Kohli, Jianuan Zhou, Florentin Constancias, Yang Liu, Zhao Cai, May Margarette Santillan Salido, Daniela I. Drautz-Moses, Scott A. Rice, Stephan Christoph Schuster, Zhao Zhi Boo, Bin Wu, Staffan Kjelleberg, Tim Tolker-Nielsen, Rajamani Lakshminarayanan, Roger W. Beuerman, Liang Yang, Michael Givskov

**Affiliations:** a Singapore Centre for Environmental Life Sciences Engineering (SCELSE), Nanyang Technological University, Singapore, Singapore; b Ocular Infections and Anti-Microbials Research Group, Singapore Eye Research Institute, Singapore, Singapore; c Department of Microbiology and Immunology, Yong Loo Lin School of Medicine, National University of Singapore, National University Health System, Singapore, Singapore; d Department of Applied Biology and Chemical Technology, The Hong Kong Polytechnic University, Hong Kong; e Forensics Genomics International (FGI), BGI-Shenzhen, Shenzhen, China; f Alfred Wegener-Institut Helmholtz-Zentrum für Polar- und Meeresforschung, Bremerhaven, Germany; g Guangdong Province Key Laboratory of Microbial Signals and Disease Control, Integrative Microbiology Research Centre, South China Agricultural University, Guangzhou, China; h Department of Health Sciences and Technology, ETH Zürich, Zurich, Switzerland; i School of Medicine, Southern University of Science and Technology, Shenzhen, Guangdong Province, China; j School of Biological Sciences, Nanyang Technological University, Singapore, Singapore; k CSIRO, Agriculture and Food, Microbiomes for One Systems Health, Canberra, Australia; l School of Biological, Earth and Environmental Sciences, University of New South Wales, Sydney, New South Wales, Australia; m Costerton Biofilm Center, Department of Immunology and Microbiology, University of Copenhagen, Copenhagen, Denmark; n Department of Pharmacy, National University of Singapore, Singapore, Singapore; o Academic Clinical Program in Ophthalmology and Visual Sciences Academic Clinical Program, Duke-NUS Medical School, Singapore, Singapore; p SRP Neuroscience and Behavioural Disorders and Emerging Infectious Diseases, Duke-NUS, Singapore, Singapore; q Ophthalmology, Yong Loo Lin School of Medicine, National University of Singapore, Singapore, Singapore; r Department of Ophthalmology, The University of Tennessee Health Science Center, Memphis, Tennessee, USA; University of Pennsylvania

**Keywords:** *Pseudomonas aeruginosa*, *in vivo* biofilms, mouse corneal infection, transcriptomics, c-di-GMP, type III secretion system, immune response

## Abstract

Pseudomonas aeruginosa is generally believed to establish biofilm-associated infections under the regulation of the secondary messenger c-di-GMP. To evaluate P. aeruginosa biofilm physiology during ocular infections, comparative transcriptomic analysis was performed on wild-type P. aeruginosa PAO1, a Δ*wspF* mutant strain (high c-di-GMP levels), and a p*_lac_*-*yhjH*-containing strain (low c-di-GMP levels) from mouse corneal infection, as well as *in vitro* biofilm and planktonic cultures. The c-di-GMP content in P. aeruginosa during corneal infection was monitored using a fluorescent c-di-GMP reporter strain. Biofilm-related genes were induced in *in vivo* PAO1 compared to *in vitro* planktonic bacteria. Several diguanylate cyclases and phosphodiesterases were commonly regulated in *in vivo* PAO1 and *in vitro* biofilm compared to *in vitro* planktonic bacteria. Several exopolysaccharide genes and motility genes were induced and downregulated, respectively, in *in vivo* PAO1 and the *in vivo* Δ*wspF* mutant compared to the *in vivo* p*_lac_-yhjH*-containing strain. Elevation of c-di-GMP levels in P. aeruginosa began as early as 2 h postinfection. The Δ*wspF* mutant was less susceptible to host clearance than the p*_lac_-yhjH*-containing strain and could suppress host immune responses. The type III secretion system (T3SS) was induced in *in vivo* PAO1 compared to *in vitro* biofilm bacteria. A Δ*wspF* mutant with a defective T3SS was more susceptible to host clearance than a Δ*wspF* mutant with a functional T3SS. Our study suggests that elevated intracellular c-di-GMP levels and T3SS activity in P. aeruginosa are necessary for establishment of infection and modulation of host immune responses in mouse cornea.

## INTRODUCTION

Corneal infections associated with contact lens wear are a common cause of ocular disease and vision loss (1–3). The Gram-negative opportunistic pathogen Pseudomonas aeruginosa is an important etiologic agent of a variety of ocular infectious diseases (4). Contact lens wearers are the primary target for these infections in many countries, such as the United Kingdom and United States (5). In addition, a recent prospective and nonrandomized study revealed that P. aeruginosa is the most common bacterial species in causing infectious keratitis across Asia (6).

Studies have suggested the presence of bacterial biofilms on the infected ocular surface and have demonstrated an enhanced biofilm formation capability of ocular bacterial clinical isolates *in vitro* (7–11). Microscopic inspections have demonstrated the presence of bacterial aggregates and biofilm matrix in experimental infections of the mouse cornea (12–15). Also, there appears to be a correlation between the bacterium’s biofilm formation ability and detrimental effects on visual acuity (16). However, whether bacteria in corneal infections display a biofilm-associated physiology still needs to be established.

Evidence has been presented that the P. aeruginosa type III secretion system (T3SS) contributes to the pathogenesis of P. aeruginosa keratitis. Thus, the T3SS activity of P. aeruginosa was shown to prevent reactive oxygen species (reactive oxygen species) production by neutrophils as well as to cause neutrophil apoptosis during corneal infection (11, 17–19). However, previous *in vitro* studies have demonstrated an inverse correlation between the expression of biofilm factors and T3SS in P. aeruginosa (20, 21), and more evidence is needed for a role of both biofilm formation and T3SS in corneal infection.

Biofilms are well known for their notorious property of resistance to antibiotic treatment (22). They are a source of many recalcitrant infections (23), and treatment resistance due to corneal biofilms for even a short period of time could lead to substantial impact on patients (i.e., vision loss). Therefore, there is a growing concern about achieving a better understanding of corneal pathogenesis and its effective treatment.

In general, biofilm formation and dispersal are processes responsive to the levels of the intracellular secondary messenger c-di-GMP, which is synthesized by diguanylate cyclase (DGC) enzymes and degraded by phosphodiesterase (PDE) enzymes (24). *In vitro* experiments suggest that an elevated intracellular c-di-GMP level represses P. aeruginosa cell motility and induces extracellular matrix synthesis, resulting in biofilm formation (25, 26). In contrast, lowering the intracellular c-di-GMP level of biofilm cells by inducing the expression of PDE enzymes was shown to disperse biofilms in both *in vitro* and *in vivo* experiments (27, 28).

Here, we adopted a mouse model of corneal infection (13, 19, 29) as an approach to study the course of infectious keratitis and the mode of growth of P. aeruginosa during corneal infection. We employed dual transcriptome sequencing (RNA-Seq) technology for comparative analysis of the transcriptomes of the *in vivo* PAO1 wild-type strain with transcriptomes of *in vitro* biofilm and planktonic cells. We also compared *in vivo* transcriptomes of the PAO1 wild type, a Δ*wspF* mutant, and a p*_lac_-yhjH*-containing strain to identify biofilm features in the P. aeruginosa wild type during keratitis. We found that P. aeruginosa contains an elevated level of c-di-GMP during corneal infection and that the bacteria express biofilm-associated factors. Moreover, we found that the T3SS is upregulated in P. aeruginosa during corneal infection. We demonstrated that high c-di-GMP levels and the T3SS system confer a benefit to P. aeruginosa during corneal infection. We also demonstrated that both the elevated intracellular c-di-GMP level and the activity of T3SS in P. aeruginosa affect the host-pathogen interactions. Hence, we propose both lowering intracellular c-di-GMP level and inactivating the activity of T3SS concurrently as a novel therapeutic approach to manage biofilm-associated P. aeruginosa keratitis.

## RESULTS

### Transcriptomes of *in vivo* P. aeruginosa cells.

To examine the *in vivo* P. aeruginosa PAO1 wild-type mode of growth (biofilm versus planktonic) and its impact on the host, we performed a dual RNA-Seq analysis on P. aeruginosa and corneal cells from the time points 2 days postinfection (dpi) and 7 dpi. The fraction of reads mapping to P. aeruginosa from 2-dpi samples ranged from 1.24% to 3.21% (see Table S2 in the supplemental material), while the corresponding fraction of reads from 7-dpi samples ranged from 0.01% to 0.30% of the total number of reads (Table S3). Due to insufficient read coverage, samples from 7-dpi corneas were discarded. The transcriptomes of the P. aeruginosa PAO1 wild type from 2-dpi corneas (*in vivo* PAO1) were compared to our previously characterized transcriptomes of *in vitro* P. aeruginosa biofilm and planktonic cells (30).

**FIG 1:**
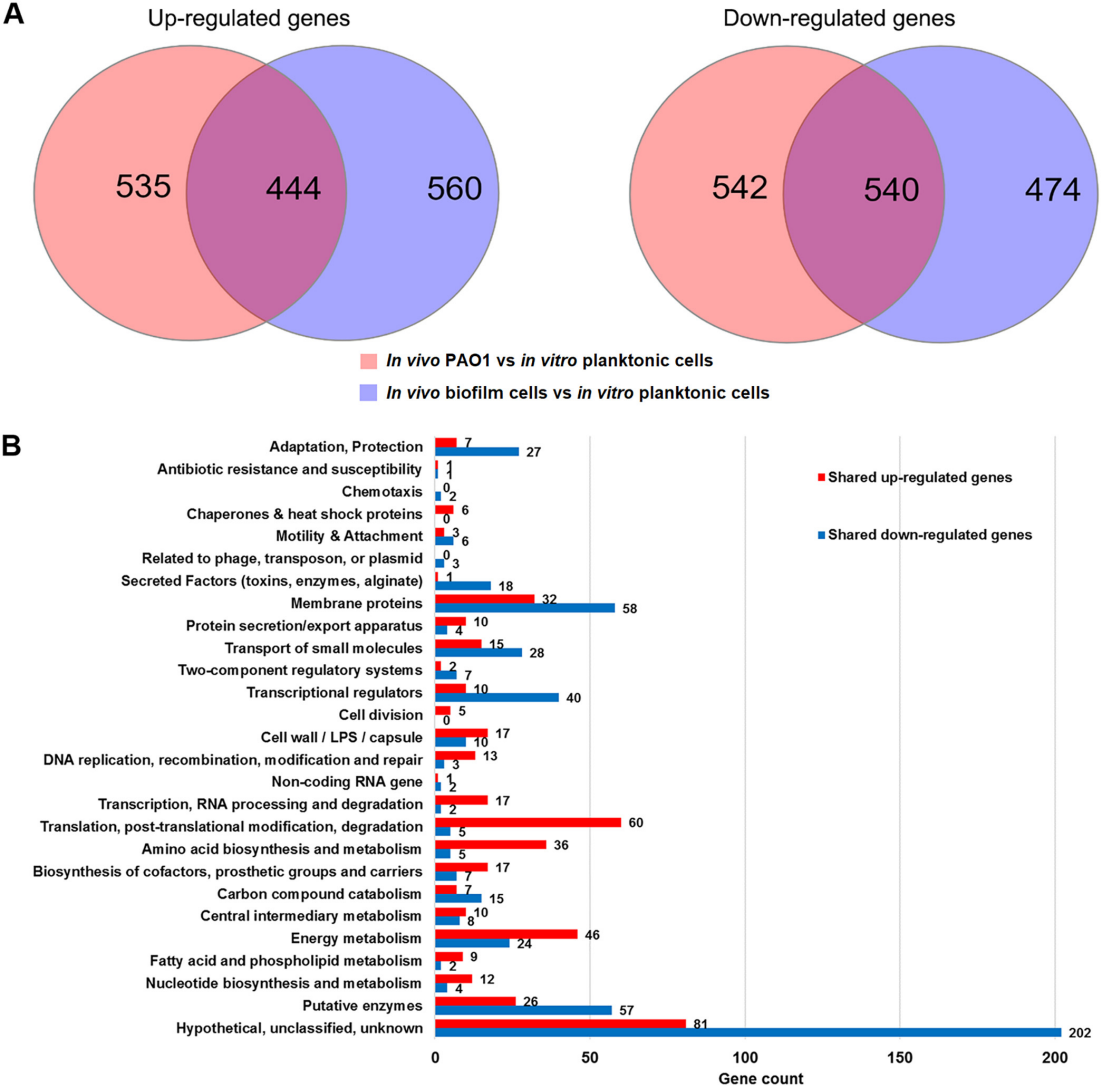
Comparative transcriptomic analysis of P. aeruginosa in experimental mouse corneal infection and under the *in vitro* condition. (A) Venn diagrams depicting the shared upregulated and downregulation genes between 2 dpi in the *in vivo* PAO1 wild-type and *in vitro* biofilm cells compared to *in vitro* planktonic cells; (B) functional enrichment analysis of shared upregulated and downregulated genes using PseudoCAP function classification.

To identify possible “biofilm signature” genes between *in vivo* PAO1 and *in vitro* P. aeruginosa biofilm cells, we compared both of their transcriptomes with those of *in vitro* planktonic cells. Using a negative binomial test with a *P* value cutoff of 0.05 and a fold change cutoff of 2, we found that 979 genes were upregulated, and 1,082 genes were downregulated in *in vivo* PAO1 compared with *in vitro* planktonic cells (Fig. 1A; Data Set S1). In addition, 1,004 genes and 1,014 genes were found upregulated and downregulated, respectively, in *in vitro* biofilm cells compared with *in vitro* planktonic cells (Fig. 1A; Data Set S2). Interestingly, there appeared to be 444 shared upregulated genes and 540 shared downregulated genes between *in vivo* PAO1 and *in vitro* biofilm cells compared to *in vitro* planktonic cells (Fig. 1A; Data Set S3). Functional enrichment analysis showed that a large number of genes involved in (i) transcription, RNA process, and degradation, (ii) translation, posttranslation modification, and degradation, and (iii) energy metabolism were induced in both *in vivo* PAO1 and *in vitro* biofilm cells compared to *in vitro* planktonic cells (Fig. 1B). Notably, further analysis of the shared upregulated and downregulated genes revealed genes involved in synthesis or degradation of c-di-GMP, as well as c-di-GMP-regulated genes. One gene encoding the GGDEF-containing protein was induced (*siaD*), while 5 genes encoding GGDEF-containing proteins (*roeA*, *gcbA*, *PA0290*, *PA1851*, and *PA4929*) were downregulated (Table 1). Five PDE-encoding genes (*rmcA*, *rbdA*, *nbdA*, *dipA*, and *PA2567*) and 1 gene encoding a GGDEF-EAL domain-containing protein (*PA1181*) were downregulated. Three genes encoding HD-GYP domain-containing proteins (*PA2572*, *PA4108*, and *PA4781*) were downregulated as well. A surface adhesion gene (*cdrA*) was upregulated, while genes involved in flagellar synthesis were downregulated (Table 1). Furthermore, genes encoding biofilm matrix components, such as Pel exopolysaccharide (e.g., *pelB*, *pelC*, *pelD*, and *pelF*) and Cup fimbriae (e.g., *cupA1* and *cupA2*), were highly expressed in *in vivo* PAO1 compared with *in vitro* planktonic cells (Data Set S1). This result is in accordance with other studies (31, 32) and was validated by quantitative reverse transcriptase PCR (qRT-PCR) analysis (Fig. S1). These results strongly suggest that the c-di-GMP level was elevated in *in vivo* PAO1.

**TABLE 1:** Shared regulated genes involved in synthesis and degradation of c-di-GMP and c-di-GMP-regulated genes from *in vivo* PAO1 and *in vitro* biofilm cells compared to *in vitro* planktonic cells

Gene no.	Gene name	Fold change for^*a*^:		Annotation^*b*^
		*In vivo* PAO1 vs *in vitro* planktonic cells	*In vitro* biofilm cells vs *in vitro* planktonic cells	
*PA0169*	*siaD*	7.0	15.4	Diguanylate cyclase
*PA1107*	*roeA*	−9.2	−3.6	Diguanylate cyclase
*PA4929*		−9.6	−4.8	Diguanylate cyclase
*PA4843*	*gcbA*	−14.3	−8.0	Diguanylate cyclase
*PA0575*	*rmcA*	−12.5	−3.4	c-di-GMP phosphodiesterase
*PA0861*	*rbdA*	−4.9	−2.4	c-di-GMP phosphodiesterase
*PA2567*		−3.6	−8.6	c-di-GMP phosphodiesterase
*PA3311*	*nbdA*	−8.4	−3.1	c-di-GMP phosphodiesterase
*PA4108*		−3.0	−5.7	c-di-GMP phosphodiesterase
*PA4781*		−5.2	−2.6	c-di-GMP phosphodiesterase
*PA5017*	*dipA*	−3.6	−2.1	c-di-GMP phosphodiesterase
*PA0290*		−2.4	−4.2	ND
*PA1181*		−3.4	−2.0	ND
*PA1851*		−2.3	−3.4	ND
*PA2572*		−13.5	−3.2	ND
*PA4625*	*cdrA*	2.0	21.6	Adhesin
*PA3351*	*flgM*	−4.8	−3.8	Anti-sigma 28 factor
*PA1080*	*flgE*	−4.2	−2.1	Flagellar hook protein FlgE
*PA1082*	*flgG*	−2.7	−2.0	Flagellar basal-body rod protein FlgG
*PA1092*	*fliC*	−2.5	−5.9	Flagellin type B
*PA1094*	*fliD*	−2.3	−3.5	Flagellar capping protein FliD

a *P* < 0.01.

b Manual annotation based on the literature and the Pseudomonas Genome Database. ND, not yet determined as diguanylate cyclase or cyclic di-GMP phosphodiesterase.

To further determine the biofilm physiology of *in vivo* PAO1, we first compared transcriptomes of the Δ*wspF* strain, a mutant that contains high levels of c-di-GMP (33), from 2-dpi corneas (*in vivo* Δ*wspF* mutant) and transcriptomes of a p*_lac_*-*yhjH*-containing strain that is depleted of c-di-GMP (34), from 2-dpi corneas (*in vivo* p*_lac_*-*yhjH*-containing strain). We noted that 46 genes were upregulated and 87 genes were downregulated in the *in vivo* Δ*wspF* mutant compared with the *in vivo* p*_lac_*-*yhjH*-containing strain (Data Set S4). Exopolysaccharide genes (*pelA*, *pelB*, *pelD*, *pslA*, and *pslB*) and *cdrA* were induced, while motility genes (*pilA*, *pilM*, *pilN*, *pilO*, *pilP*, *pilQ*, *pilV*, *fimV*, *fimU*, *flgH*, and *flgK*) were downregulated in the *in vivo* Δ*wspF* mutant compared to the *in vivo* p*_lac_*-*yhjH-*containing strain. Moreover, 49 and 79 genes were noted as up- and downregulated, respectively, in *in vivo* PAO1 compared to the *in vivo* p*_lac_*-*yhjH*-containing strain (Data Set S5). The comparison revealed that the exopolysaccharide gene *pslA* was induced, and motility genes (*pilA*, *pilN*, *pilO*, *pilP*, *pilQ*, *pilV*, and *fimV*) were downregulated in *in vivo* PAO1 compared to the *in vivo* p*_lac_*-*yhjH*-containing strain. The statistics of the differentially expressed genes for each transcriptomic comparison group are shown in Table S4. Taken together, the common upregulation of exopolysaccharide genes and downregulation of motility genes in both comparisons further support the notion that the c-di-GMP level was elevated in the PAO1 wild type during corneal infections.

### c-di-GMP levels are elevated in P. aeruginosa during corneal infections.

**FIG 2:**
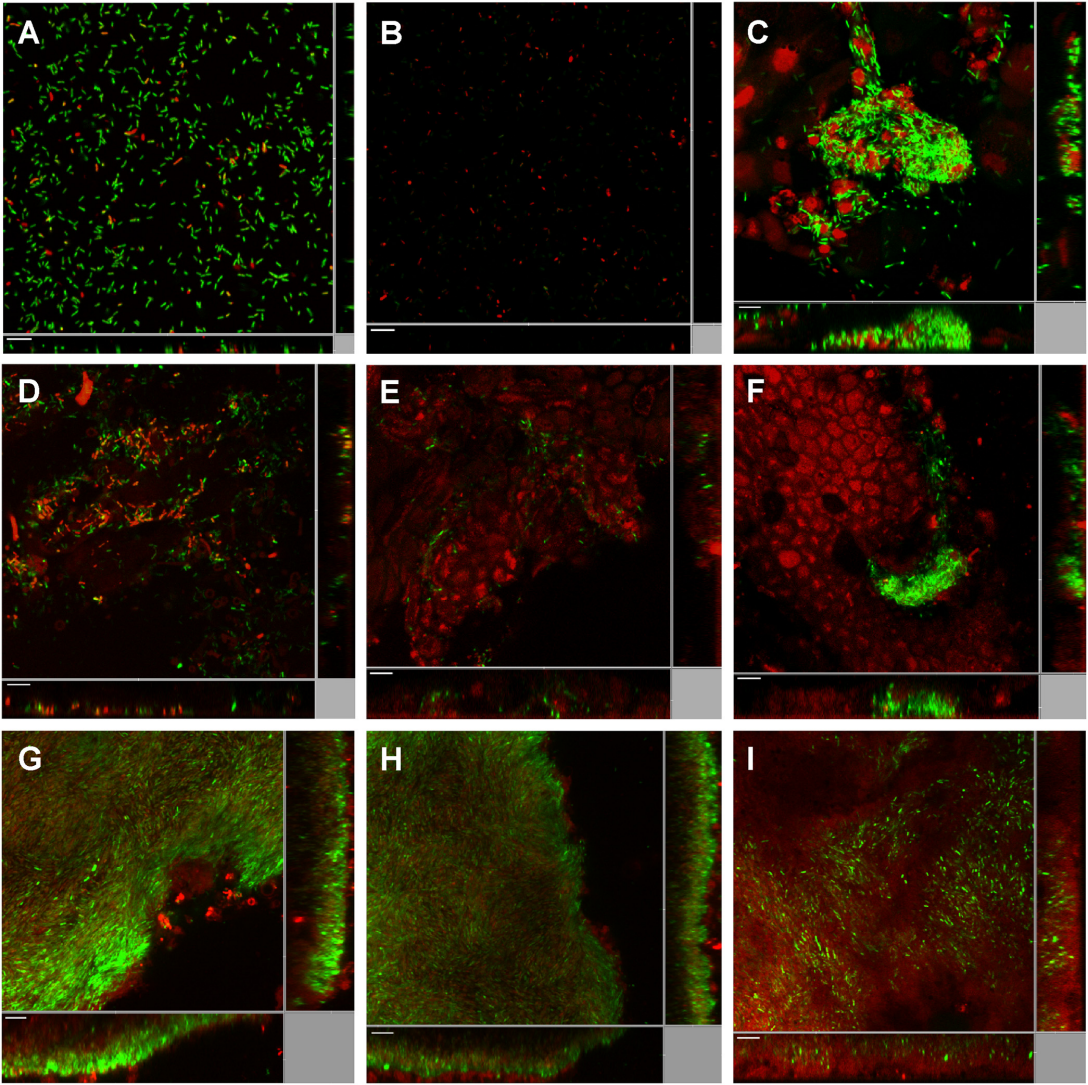
Induction of the c-di-GMP reporter fusion p*_cdrA_*-*gfp* in P. aeruginosa during mouse corneal infection. (A) Planktonic PAO1/p*_lac_*-*gfp* cells; (B) planktonic PAO1/p*_cdrA_*-*gfp* cells; (C) PAO1/p*_lac_*-*gfp* cells at 8 h postinfection (8 hpi) of mouse cornea; (D) PAO1/p*_cdrA_*-*gfp* cells at 2 hpi of mouse cornea; (E) PAO1/p*_cdrA_*-*gfp* cells at 4 hpi of mouse cornea; (F) PAO1/p*_cdrA_*-*gfp* cells at 8 hpi of mouse cornea; (G) PAO1/p*_cdrA_*-*gfp* cells at 1 dpi of mouse cornea; (H) PAO1/p*_cdrA_*-*gfp* cells at 4 dpi of mouse cornea; (I) PAO1/p*_cdrA_*-*gfp* cells at 7 dpi of mouse cornea. SYTO 62 was used to stain host cells as well as P. aeruginosa cells lacking fluorescence. Green fluorescence represents constitutive expression of p*_lac_*-*gfp* (A and C) and expression of the p*_cdrA_-gfp* reporter fusion (B and D to I), and red fluorescence represents SYTO 62 staining. Experiments were performed in triplicate, and a representative image for each condition is shown. Scale bars, 10 μm.

To further address whether the intracellular c-di-GMP level in P. aeruginosa is elevated during corneal infection, we used P. aeruginosa carrying a p*_cdrA_*-*gfp* gene fusion, which is a well-established c-di-GMP reporter strain (34–36). In the planktonic state, the c-di-GMP reporter bacteria exhibited an extremely low expression level of the p*_cdrA_*-*gfp* fusion (below the detection limit) compared with a P. aeruginosa strain that carries a p*_lac_-gfp* fusion and constitutively expresses the green fluorescent protein (GFP) gene *gfp* (Fig. 2A and B). This indicates that P. aeruginosa bacteria in the planktonic state contain a low level of c-di-GMP, which is in agreement with previous work (35). The p*_lac_-gfp*-tagged strain showed a biofilm-like aggregation morphology at 8 h postinfection (hpi) in our mouse model of corneal infection (Fig. 2C). We monitored the expression of the p*_cdrA_*-*gfp* fusion in the c-di-GMP reporter strain at 2 hpi (Fig. 2D), 4 hpi (Fig. 2E), and 8 hpi (Fig. 2F). GFP expression was induced in subpopulations of the bacterial cells as early as 2 hpi (Fig. 2D), suggesting that the c-di-GMP level starts to elevate shortly after the establishment of corneal infection. At 4 hpi and 8 hpi, small biofilm-like aggregates with bright GFP fluorescent signals were visualized in the reporter strain-infected corneas (Fig. 2E and F). Expression of the p*_cdrA_*-*gfp* reporter fusion was maintained at a relatively high level at 2 dpi and 4 dpi, suggesting a high c-di-GMP level in the P. aeruginosa cells during the course of corneal infection (Fig. 2G and H). Interestingly, expression of the p*_cdrA_*-*gfp* fusion was reduced in P. aeruginosa at 7 dpi (Fig. 2I), suggesting that either the intracellular c-di-GMP level had reduced or that most of the bacterial cells had become nonviable. As a control, green fluorescence was not detected in uninfected scratched mouse corneas (Fig. S2A and B).

To identify the potential DGCs responsible for the elevated intracellular c-di-GMP level in P. aeruginosa during corneal infections, we examined the expression of p*_cdrA_*-*gfp* in *siaD* and *PA5442* (an uncharacterized gene encoding a GGDEF domain-containing protein) mutants during corneal infection. This is because these DGC-encoding genes were induced in *in vivo* PAO1 at 2 dpi by 7- and 2.6-fold, respectively, compared with *in vitro* planktonic cells (Data Set S1). However, during corneal infection, the p*_cdrA_*-*gfp* fusion gene was expressed at similar levels in these mutants to that in the wild type (Fig. S3). This result suggests that the high level of c-di-GMP in P. aeruginosa during corneal infection was not solely due to the activities of these two DGCs.

Comparing the transcriptomes of *in vivo* PAO1 with those of *in vitro* planktonic cells, we noticed that the expression of a few PDE-encoding genes—*dipA* (*PA5017*), *mucR* (*PA1727*), *nbdA* (*PA3311*), *PA0575*, *PA1181*, and *PA2072*—was downregulated in *in vivo* PAO1 compared with *in vitro* planktonic cells (Data Set S1). This was validated by qRT-PCR analysis (Fig. S1). The downregulation of these PDE-encoding genes may account for the elevated content of c-di-GMP in the P. aeruginosa bacteria during corneal infection.

### c-di-GMP is required for the establishment of microcolonies of P. aeruginosa during corneal infection.

**FIG 3:**
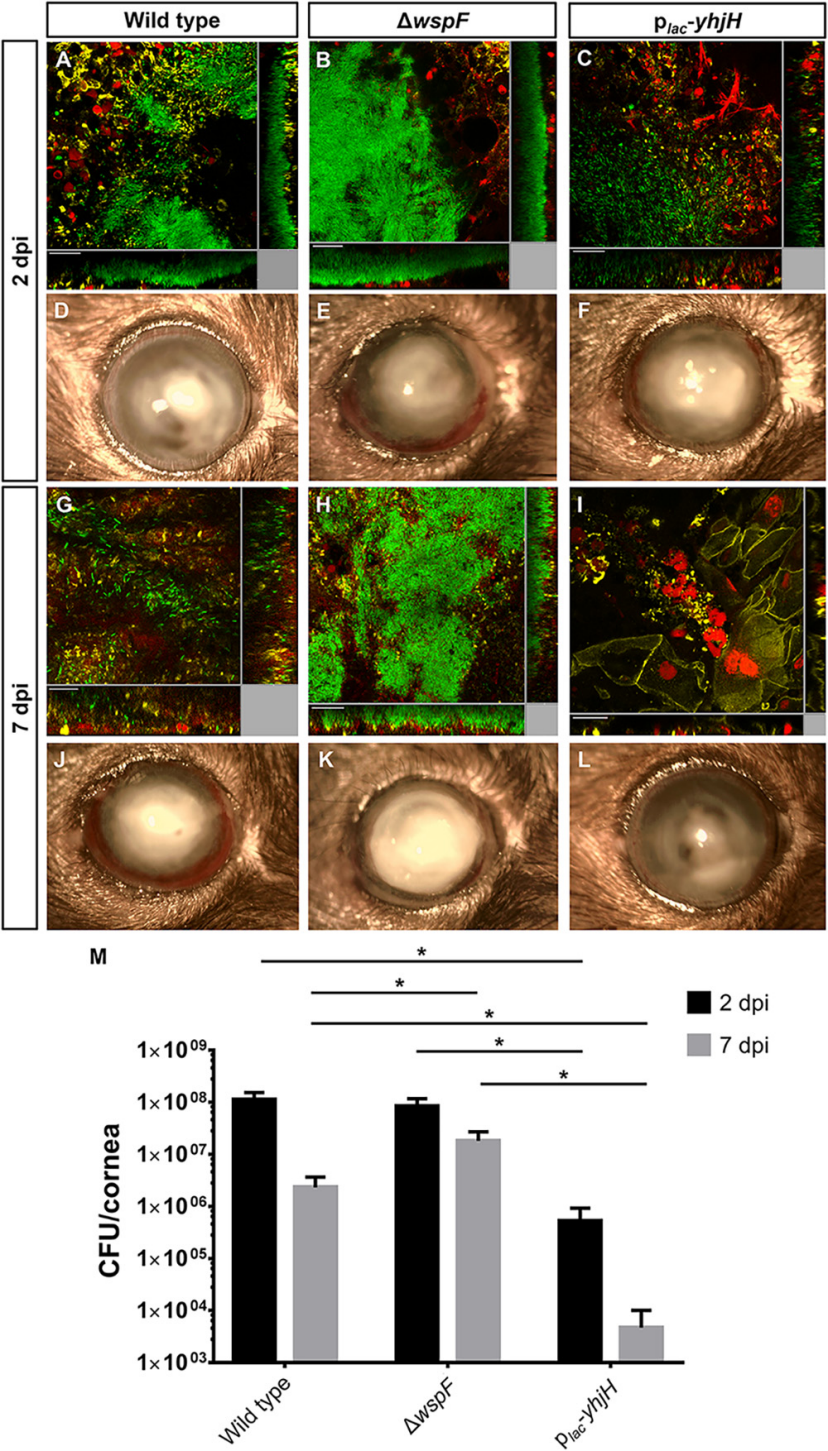
Bacterial load of P. aeruginosa in mouse cornea after infection with the wild-type PAO1, Δ*wspF* mutant, and p*_lac_*-*yhjH* strains at 2 dpi and 7 dpi. (A to C and G to I) Confocal images of infected mouse cornea with the PAO1 wild-type, Δ*wspF*, and p*_lac_*-*yhjH* strains at 2 dpi and 7 dpi. The P. aeruginosa bacteria were tagged with p*_lac_*-*gfp* in a mini-Tn7 construct. The red fluorescence represents the staining of lysosomes by LysoTracker Red DND-99. The yellow fluorescence represents Alexa Fluor 635-phalloidin, which stains F-actin of the eukaryotic cells. (D to F and J to L) Slit lamp images of infected mouse cornea with the wild-type PAO1, Δ*wspF*, and p*_lac_*-*yhjH* strains at 2 dpi and 7 dpi. Experiments were performed in triplicate, and a representative image of each condition is shown. Scale bars, 20 μm. (M) CFU of corneas infected with the wild-type PAO1, Δ*wspF*, and p*_lac_*-*yhjH* strains at 2 dpi and 7 dpi. Mean values and SD from triplicate experiments are shown. *, *P* < 0.01, Student's *t* test.

The comparative transcriptomic analysis and c-di-GMP reporter fusion experiments strongly indicated that the c-di-GMP level in P. aeruginosa is elevated during corneal infection. Accordingly, we investigated if an elevated intracellular c-di-GMP level is essential for microcolony formation and contributes to P. aeruginosa resistance to attack by the host immune system during corneal infection. We infected the mouse corneas with the *gfp*-tagged P. aeruginosa PAO1 wild type, Δ*wspF* mutant, and p*_lac_*-*yhjH*-containing strain and monitored the establishment of microcolonies of these strains at 2 dpi and 7 dpi. To visualize the interactions between the host cells and P. aeruginosa cells, the LysoTracker Red DND-99 stain was used to identify lysosome and acidic organelles, while Alexa Fluor 635-conjugated phalloidin was used to stain the F-actin of eukaryotic cells. Via confocal imaging, it was observed that wild-type P. aeruginosa and Δ*wspF* mutant bacteria formed large and densely packed microcolonies at the infection site and were surrounded by host cells at 2 dpi (Fig. 3A and B). While large cell aggregates were still observable in Δ*wspF*-infected cornea at 7 dpi (Fig. 3H), small cell aggregates were detected in wild-type P. aeruginosa*-*infected cornea (Fig. 3G). Contrarily, the p*_lac_*-*yhjH*-containing strain formed irregular, small cell aggregates at the infection sites at 2 dpi (Fig. 3C). For the infection with the p*_lac_*-*yhjH*-containing strain, a large number of host cells accumulated at 7 dpi, and few visible and viable bacteria were present (Fig. 3I). Through slit lamp microscopy, an increased corneal opacity and hypopyon were observed on all 2-dpi samples (Fig. 3D to F) compared to uninfected corneas (Fig. S2C). However, drastic increases in corneal opacity and hypopyon were noted in wild-type (Fig. 3J) and Δ*wspF* strain-infected (Fig. 3K) cornea at 7 dpi, while significant less corneal opacity and hypopyon were observed in cornea infected with the p*_lac_*-*yhjH*-containing strain (Fig. 3L). The bacterial loads of the wild-type, Δ*wspF*, and p*_lac_*-*yhjH-*containing strains per cornea at 2 dpi were approximately 1 × 10^8^, 1 × 10^8^, and 5 × 10^5^ cells, respectively (Fig. 3M). Whereas, at 7 dpi, the bacterial loads of the wild-type, Δ*wspF*, and p*_lac_*-*yhjH-*containing strains were approximately 2 × 10^6^, 2 × 10^7^, and 4 × 10^3^ cells, respectively (Fig. 3M). The bacterial loads of P. aeruginosa strains at 2 dpi and 7 dpi correlated well with both confocal microscopy and slit lamp microscopy imaging analysis, which strongly suggests that high intracellular c-di-GMP levels contribute to P. aeruginosa resistance to host immune attack during corneal infection.

### Elevated c-di-GMP level in P. aeruginosa modulates the host immune response.

**FIG 4:**
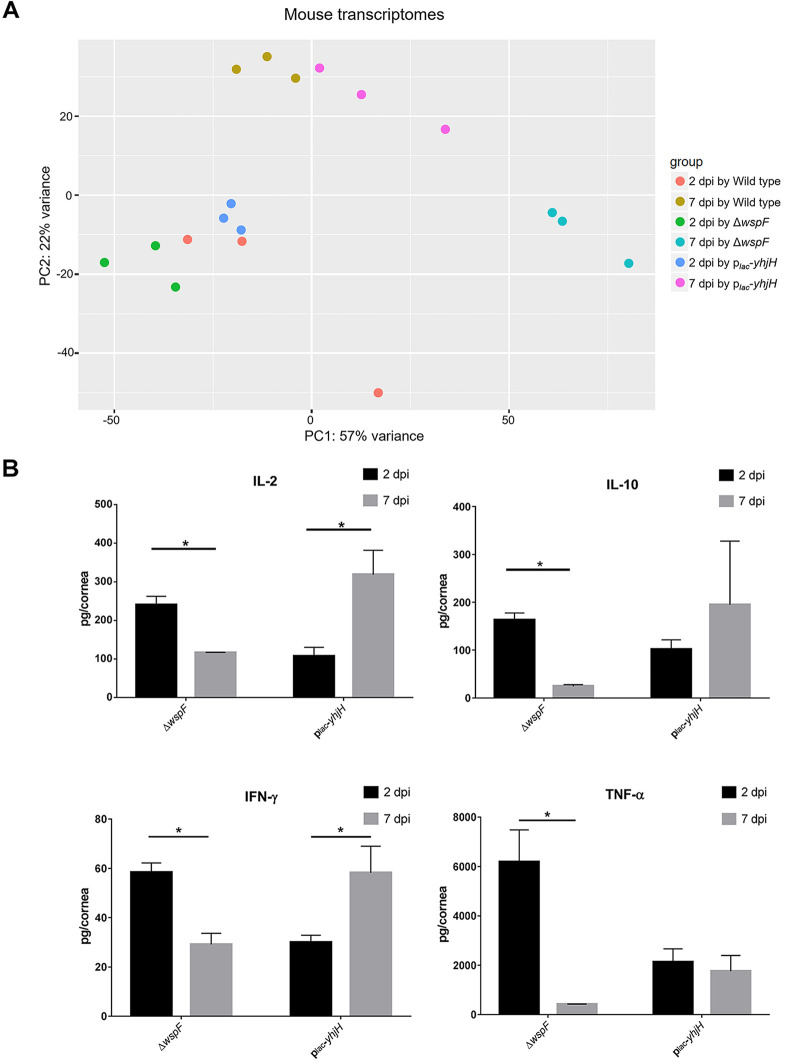
Transcriptomes and immune responses of mouse corneal cells at 2 dpi and 7 dpi with the P. aeruginosa wild-type PAO1, Δ*wspF*, and p*_lac_*-*yhjH* strains. (A) PCA of the infected mouse corneal cells. Raw RNA-Seq data were normalized using the DESeq package before PCA. (B) Production of cytokines by mouse corneal cells at 2 dpi and 7 dpi with P. aeruginosa Δ*wspF* and p*_lac_*-*yhjH* strains. Black and gray bars represent 2 dpi and 7 dpi, respectively. Mean values and SD from triplicate experiments are shown. *, *P* < 0.05, Student's *t* test.

Biofilm formation has been proposed to shift bacterial infections from an acute phase to a chronic phase since biofilm cells can evade host immune attack (37). To examine the host responses toward P. aeruginosa infection, we examined the host-associated RNA reads using a dual RNA-Seq data set to perform a comparative transcriptomic analysis of mouse corneas infected by the P. aeruginosa wild-type, Δ*wspF*, and p*_lac_*-*yhjH*-containing strains at 2 dpi and 7 dpi. Totals of 68.18% to 72.05% of the total reads from 2 dpi mapped to the mouse genome, while 76.44% to 79.75% of the total reads from 7 dpi mapped to the mouse genome (Table S2). The projection of the host transcriptomes by principal-component analysis (PCA) revealed that P. aeruginosa infections affected host gene expression in a time-dependent manner, and profiles by all strains clustered separately from each other (Fig. 4A). The distinct clustering of profiles can be explained by differing c-di-GMP content in each strain. The Δ*wspF* strain is “locked” in a high-c-di-GMP-content state, while the p*_lac_*-*yhjH*-containing strain is “locked” in a low-c-di-GMP-content state, and the c-di-GMP content in the wild-type is dynamic and change in response to its environment.

To further investigate the host response toward P. aeruginosa with high c-di-GMP levels, we compared the host transcriptomes at 2 dpi and 7 dpi by infection with the Δ*wspF* mutant. Using a *P* value cutoff of 0.05 and a fold change cutoff of 5, 1,385 host genes were upregulated and 507 host genes were downregulated in corneal cells at 7 dpi compared to corneal cells at 2 dpi caused by the Δ*wspF* mutation (Data Set S6). The functional enrichment for the biological process illustrated the downregulation of host immune response in 7-dpi compared to 2-dpi Δ*wspF* mutant-infected corneas (Fig. S4). Transcriptomic data revealed that genes involved in the host immune response toward bacterial infections were downregulated at 7 dpi compared to 2 dpi caused by the Δ*wspF* mutation. Genes involved in inflammation, such as *IL-10* (encoding interleukin-10 [IL-10]), *IL-23a* (encoding interleukin 23, alpha subunit), *tnf* (encoding tumor necrosis factor [TNF]), and *ifnlr1* (encoding interferon lambda [IFN-λ] receptor 1), were downregulated by 21.4-fold, 20.4-fold, 18.4-fold, and 9.8-fold, respectively (Data Set S6), in the samples obtained from Δ*wspF* strain-infected corneas.

To validate the transcriptomic findings, cytokine quantifications were carried out on Δ*wspF* strain-infected and p*_lac_*-*yhjH* strain-infected corneas for 2 and 7 dpi. In agreement with the transcriptomic analysis, cytokines IL-2, IL-10, IFN-γ, and TNF-α were induced at 2 dpi in response to both P. aeruginosa strains (Fig. 4B). These cytokines were reduced at 7 dpi in corneas infected with the Δ*wspF* mutant (Fig. 4B), while they remained at the same levels or increased at 7 dpi in corneas infected with the c-di-GMP-depleted strain (Fig. 4B). The reduced cytokine levels in mouse corneas infected with the Δ*wspF* mutant at 7 dpi compared with 2 dpi corresponded well with less host cells associated with the Δ*wspF* microcolonies at 7 dpi (Fig. 3H). In contrast, the high cytokine levels in mouse corneas infected with the P. aeruginosa c-di-GMP-depleted p*_lac_*-*yhjH* strain at 7 dpi compared with 2 dpi corresponded well with the large numbers of host cells at the sites of infection at 7 dpi (Fig. 3I). The cytokine levels have been previously reported to peak at early stages (1 to 3 dpi) of P. aeruginosa mouse corneal infection and then decline to baseline levels in BALB/c mice and remain at lower levels in C57BL/6 mice at the late stage of corneal infection (5 to 7 dpi) (38). Our study showed that the increased c-di-GMP level in P. aeruginosa was associated with a reduction of cytokine levels in the infected corneas of C57BL/6 mice.

### The activity of the type III secretion system is critical to P. aeruginosa survival during corneal infection.

Transcriptomic analysis of the *in vivo* PAO1 and the *in vitro* planktonic cells revealed the induction of P. aeruginosa T3SS activity (*pscF*, *pscG*, *pscI*, *pscK*, and *pscO*) during corneal infection (Data Set S1). Furthermore, genes involved in the T3SS such as *exoT*, *pcrV*, and *pscJ* were highly induced by 28.5-fold, 48.1-fold, and 15.2-fold in *in vivo* PAO1 compared with the transcriptome of *in vitro* biofilm cells (Data Set S7). To validate this finding, we used a P. aeruginosa strain containing a p*_exoT_-gfp* reporter for activity of T3SS to establish infection and to cultivate biofilm *in vitro* (39). The p*_exoT_-gfp* expression was highly induced in the reporter strain during the corneal infection compared to *in vitro* biofilm cells (Fig. S5). These results agree with previous findings (17, 19, 29) and further emphasize that the P. aeruginosa T3SS contributes to the establishment of P. aeruginosa keratitis.

**FIG 5:**
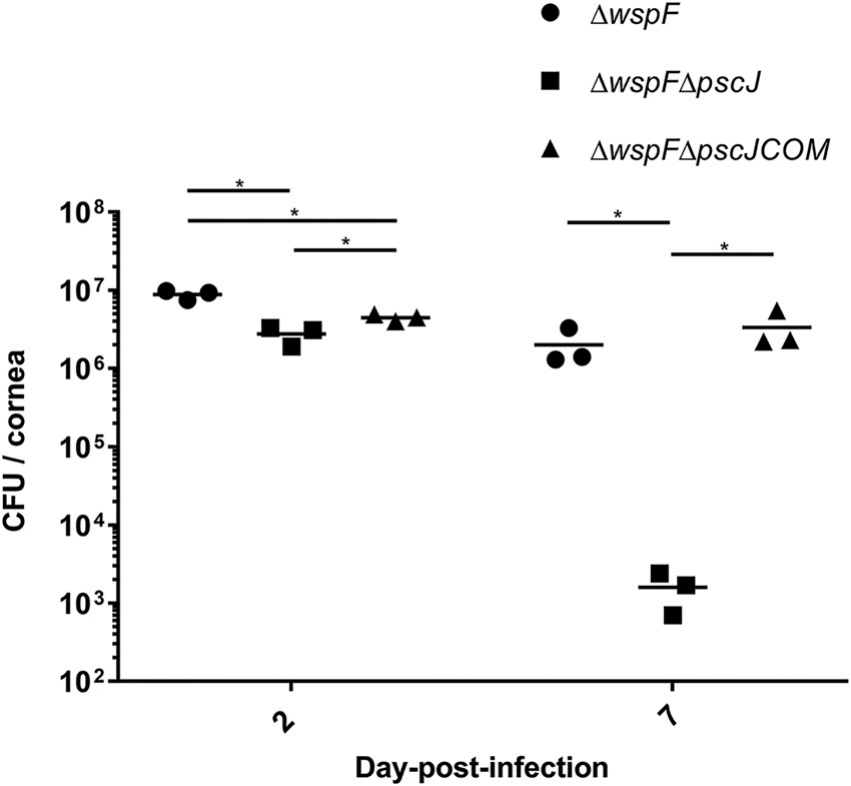
Bacterial loads in mouse cornea at 2 dpi and 7 dpi. Mouse corneas were infected with the P. aeruginosa Δ*wspF*, Δ*wspF* Δ*pscJ*, and Δ*wspF* Δ*pscJ*COM strains. The graphs depict the bacterial load (CFU) of each strain at 2 dpi and 7 dpi. Mean values and SD from triplicate experiments are shown. *, *P* < 0.05, Student's *t* test.

We then investigated the role of T3SS activity in contributing to the establishment of infection while the P. aeruginosa cells are in a biofilm mode of growth. To this end, we infected the mouse cornea with the Δ*wspF*, Δ*wspF* Δ*pscJ* (T3SS-deficient Δ*wspF* mutant), or Δ*wspF* Δ*pscJ*COM (T3SS-complemented *wspF* mutant) strain. All of these strains have a high intracellular c-di-GMP level and only differ in their T3SS activity. The bacterial loads in Δ*wspF* Δ*pscJ* strain-infected corneas were about 2 times lower than the bacterial load in Δ*wspF* strain-infected corneas at 2 dpi, while the bacterial load was approximately 1,000 times lower in Δ*wspF* Δ*pscJ* strain-infected corneas than in Δ*wspF* strain-infected corneas at 7 dpi (Fig. 5). This result suggests a significant contribution of T3SS activity to virulence even when the P. aeruginosa cells are in the biofilm mode of growth during corneal infection.

## DISCUSSION

P. aeruginosa is one of the most common microorganisms for causing keratitis in humans. Morphology-based approaches including electron microscopy and fluorescence-based techniques have previously demonstrated the presence of bacterial cell aggregates on animal cornea infected by P. aeruginosa (13, 40–42). Studies also demonstrated enhanced biofilm formation capability and virulence expression of P. aeruginosa clinical isolates that were obtained from patients’ corneas compared to laboratory strains (43, 44). However, there is still a lack of characterization of the physiology of P. aeruginosa during corneal infection for better understanding of host-pathogen interactions as well as the possibility of identifying potential new antimicrobial drug targets.

We report here that P. aeruginosa wild-type cells from experimental mouse corneal infection and *in vitro*-grown P. aeruginosa bacteria have distinct transcriptomic profiles indicative of fundamental differences in cell physiologies (Fig. 1 and Table 1), which prompted us to perform *in vivo* physiological characterization studies that allowed us to investigate the biofilm features of P. aeruginosa during corneal infection. The *in vitro* P. aeruginosa biofilm features *pel* exopolysaccharide and *cupA* fimbrial genes, which are regulated by c-di-GMP signaling and which were significantly upregulated in *in vivo* P. aeruginosa wild-type cells compared to *in vitro* planktonic cells (see Data Set S1 in the supplemental material). Moreover, our findings showed that several DGC and PDE genes displayed distinct regulation in the *in vivo* PAO1 wild type and *in vitro* biofilm compared to *in vitro* planktonic cells (Table 1; Data Set S3). We included Δ*wspF* and p*_lac_*-*yhjH* strains in our mouse corneal infections. The Δ*wspF* strain has a high c-di-GMP level due to a hyperactive WspR DGC, whereas the p*_lac_*-*yhjH*-containing strain has a low c-di-GMP level due to expression of the strong PDE YhjH. Via transcriptomic analysis, the common induction of exopolysaccharide genes and downregulation of motility genes were observed in both the *in vivo* Δ*wspF* mutant and the *in vivo* PAO1 wild type compared to the *in vivo* p*_lac_*-*yhjH*-containing strain (Data Sets S4 and 5), which further supports that PAO1 wild-type cells express biofilm features during keratitis.

We provided evidence that cornea-infecting P. aeruginosa strains assume the biofilm life-mode as judged from elevated intracellular levels of c-di-GMP; a well-described condition of biofilm development under *in vitro* conditions. A P. aeruginosa c-di-GMP bioreporter strain displayed an elevated level of c-di-GMP during corneal infection as early as 2 hpi and maintained its high c-di-GMP levels until 7 dpi (Fig. 2). Taken together, these findings indicate that P. aeruginosa keratitis is a biofilm infection, with biofilm-associated characteristics such as resistance to host immune responses and antimicrobial agents, which may eventually lead to chronic inflammatory cascades and blindness.

To understand how the c-di-GMP level in P. aeruginosa affects the establishment of infection and host responses during corneal infection, we included Δ*wspF* and p*_lac_*-*yhjH* strains in the mouse corneal infections. The densely packed microcolonies of the P. aeruginosa wild type and the Δ*wspF* mutant were clearly surrounded by host cells (Fig. 3B and H), a phenomenon also observed in P. aeruginosa chronic lung infection of cystic fibrosis patients (42, 45). The p*_lac_*-*yhjH-*containing strain was more susceptible to host clearance than the Δ*wspF* strain (Fig. 3M), which is in agreement with earlier studies suggesting that an increased intracellular c-di-GMP level protects P. aeruginosa bacteria against reactive oxygen species (ROS), while P. aeruginosa bacteria with low intracellular c-di-GMP level are more susceptible to ROS (46). The mouse transcriptome profiles of the Δ*wspF* strain-infected cornea and p*_lac_*-*yhjH*-infected cornea differed greatly (Fig. 4A), and the levels of cytokine production were lower in 7-dpi Δ*wspF* strain-infected cornea than in 7-dpi p*_lac_*-*yhjH* strain-infected cornea (Fig. 4B). Reduced production of cytokines in 7-dpi Δ*wspF* strain-infected cornea most likely plays an important role for the high persistence of infections caused by bacteria with high c-di-GMP levels. There have been studies of c-di-GMP-regulated pathogenesis in other systems (47); however, in the literature, studies of the role of c-di-GMP in corneal infection are lacking. We corroborate that P. aeruginosa bacteria in keratitis likely possess a high content of c-di-GMP that promotes persistent biofilm pathogenesis and reduced production of cytokines. The efficiency of bacterial clearance by the host could also be affected by activation of the cyclic GMP-AMP synthase stimulator of interferon gene (cGAS-STING) pathway, which has been reported to be involved in protecting the host by activating the innate immune response during bacterial infection (48–50). Apart from bacterial DNA, cyclic dinucleotides like c-di-GMP and c-di-AMP can also activate cGAS, which in turn triggers STING to initiate the host immune response (51–53). K. Chen et al. demonstrated that STING promoted host resistance against P. aeruginosa keratitis in a mouse infection model by restricting corneal inflammatory response and bacterial killing (54). Though our transcriptomic data did not reveal any differential expression of cGAS and STING genes during comparative analysis, our findings are in accordance with those of Chen et al. as we noted TNF-α production was lower in p*_lac_*-*yhjH*-containing strain-infected corneal cells than in Δ*wspF* strain-infected corneal cells at 2 dpi (Fig. 4B), and the bacterial load of the p*_lac_*-*yhjH*-containing strain was also significantly lower than that of the Δ*wspF* strain in 2 dpi (Fig. 3M). These results may indicate the increased activity of the cGAS-STING-signaling pathway in p*_lac_*-*yhjH*-containing strain-infected corneal cells compared to Δ*wspF* strain-infected corneal cells. However, more studies are required to examine the level of cGAS-STING signaling during keratitis caused by the Δ*wspF* and p*_lac_-yhjH* strains, to assess whether intracellular c-di-GMP contents in P. aeruginosa play a role in modulating the host cGAS-STING signaling activity.

Many studies have demonstrated that environmental factors can induce the activities of DGCs, leading to an elevation of the bacterial c-di-GMP level (55–57). We found that the expression of the DGC genes *siaD* and *PA5442* was enhanced in P. aeruginosa during corneal infection, but this was not deemed critical as the c-di-GMP level was still elevated in *siaD* and *PA5442* mutants under these conditions (Fig. S3). The expression of several PDE genes was downregulated under *in vivo* conditions (Fig. S1 and Data Set S1), which could likely contribute to the elevated c-di-GMP levels in the bacterial cells during corneal infection. However, our study did not to rule out the possibility of complementary effects contributed by other DGCs in maintaining the high c-di-GMP levels in the absence of *siaD* and *PA5442*, as there are over 30 genes coding for proteins containing conserved DGC motifs in P. aeruginosa (58).

P. aeruginosa in a planktonic single-cell state has been associated with acute infections, while bacteria in the biofilm state have been associated with chronic infections. The model of reciprocally regulated gene expression associated with acute and chronic infections via the RetS master regulator is well established by several groups (20, 21). The type III secretion system (T3SS) is one of the major virulence determinants in P. aeruginosa that contribute to establishment of acute infection (59). However, in our study, occurrence of both T3SS activity and biofilm physiology during corneal infection seems to contradict the established model. However, a similar phenomenon was previously noted by Fleiszig and coworkers when P. aeruginosa was treated with human tear fluid after being grown on the surface of contact lenses (11). Moreover, other studies also support the notion that T3SS activity and biofilm physiology are not mutually exclusive in P. aeruginosa infection (41, 60). However, the mechanism underlying triggering of both acute and chronic infection features in P. aeruginosa concurrently during corneal infection remains unclear. A significant reduction in bacterial load of a Δ*wspF* Δ*pscJ* mutant was noted compared to the Δ*wspF* strain. This further emphasized the importance of T3SS activity, even though the P. aeruginosa cells adopt a biofilm lifeform during corneal infection.

A number of studies have proposed c-di-GMP signaling as an antimicrobial target to eradicate biofilms (27, 61–63), and some studies have also proposed targeting of T3SS to combat corneal infection caused by P. aeruginosa (64–66). However, these concepts have not been utilized for treatment of P. aeruginosa keratitis as the physiological state of P. aeruginosa cells has been largely unknown. With strong evidence that P. aeruginosa bacteria adopt a biofilm life-form and induce T3SS activity during corneal infection, we propose targeting of both c-di-GMP signaling and T3SS activity for effective management of corneal infections caused by P. aeruginosa.

## MATERIALS AND METHODS

### Animal use.

All animal experiments were conducted in compliance with the ARVO statement for the Use of Animals in Ophthalmic and Vision Research, the *Guide for the Care and Use of Laboratory Animals* (67) under SingHealth Institutional Animal Care and Use Committee (IACUC) protocol no. 2014/SHS/901, SingHealth Institutional Biosafety Committee (IBC) approval no. SHSIBC-2014-015, and under the supervision of SingHealth Experimental Medical Centre (SEMC).

### Bacterial strains and culture media.

The bacterial strains (see Table S1 in the supplemental material) were routinely cultivated in Miller’s Luria-Bertani (LB) broth (BD Difco; USA). To construct green fluorescent protein-tagged strains, the mini-Tn*7*-Gm-p*_lac_-gfp* fusion was inserted into the chromosomes of PAO1 wild type, PAO1 Δ*wspF*, and PAO1/p*_lac_-yhjH* strains by four-parental mating using pBF13 and pRK600 vectors, as described previously (68, 69). For marker selection in P. aeruginosa, 30 μg mL^−1^ gentamicin (Gm), 50 μg mL^−1^ tetracycline (Tc), 100 μg mL^−1^ streptomycin (Strep), or 200 μg mL^−1^ carbenicillin (Cb) was used, as appropriate.

### Mouse corneal infection.

The mouse corneal infection procedures were carried out as previously described (13). Female wild-type C57BL/6 mice (7 to 8 weeks old) from The Jackson Laboratory were used in this study. Mice were anesthetized subcutaneously with 100 mg kg^−1^ ketamine and 10 mg kg^−1^ xylene and placed under a stereoscopic microscope. A sterile miniblade (Beaver-Visitec International, MA, USA) was used to make corneal scratches (*n *= 3, each 1 mm long) that did not breach the superficial stroma on the right eye, while the left eye remained untouched (70). Ten microliters of a bacterial suspension containing 1 × 10^5^ CFU/μL P. aeruginosa was topically applied to the scratched cornea.

### Visualization of c-di-GMP level in Pseudomonas aeruginosa during infection.

Procedures were carried out as mentioned previously (35). Briefly, the scratched corneas were infected with green fluorescent protein (GFP)-tagged P. aeruginosa PAO1/p*_cdrA_*-*gfp* or PAO1/p*_lac_*-*gfp* for observation of GFP expression during infection. Mice were sacrificed before the infected corneas were dissected at the time points 2 h postinfection (hpi), 4 hpi, and 8 hpi, as well as at 1 day postinfection (dpi), 2 dpi, 4 dpi, and 7 dpi. Corneas were stained with 20 μL of 5 μM SYTO 62 Red fluorescent nucleic acid stain (Thermo Fisher Scientific, Singapore), which stains nucleic acids in both bacterial and corneal cells, for 15 min before visualization with Zeiss LSM780 confocal laser scanning microscope (CLSM) (Carl Zeiss, Jena, Germany). For controls, PAO1/p*_cdrA_*-*gfp* and PAO1/p*_lac_*-*gfp* cells were cultivated in LB broth with shaking (200 rpm) at 37°C until mid-log phase before observation under the CLSM using the same parameters as viewing of dissected cornea.

### Construction of the P. aeruginosa mutants.

The upstream and downstream sequences of *PA5442* were obtained from the Pseudomonas genome database (http://www.pseudomonas.com/), and the primers used for amplification of *PA5442* are listed in Table S1. Briefly, the up and down flanking fragments of *PA5442* were amplified with primer pairs PA5442-1 and PA5442-2 and PA5442-3 and PA5442-4, respectively. The PCR products were purified and assembled with BamHI- and HindIII-digested pK18 (Gm^r^) plasmid using Gibson assembly master mix (NEB) and then transformed into Escherichia coli DH5α competent cells. Positive transformants were picked and grown in LB broth supplemented with 30 μg mL^−1^ Gm. The in-frame deletion mutant was generated by triparental conjugation with the aid of RK600 (71). Mutants were confirmed by amplification using primers PA5442-F and PA5442-R and sequencing. All other mutants were generated in the same manner. The Pseudomonas aeruginosa Δ*pscJ* mutant was constructed by a similar approach using the primers listed in Table S1. The wild-type *pscJ* gene was first cloned to the pUC18Not vector using the pscJCOM-F and pscJCOM-R primers listed in Table S1 and next cloned to the mini-Tn*7*-strep vector (68) after NotI digestion for complementation of the Δ*pscJ* mutant.

### Establishment of microcolonies and immune response characterization.

Scratched corneas were infected with the p*_lac_*-*gfp*-tagged P. aeruginosa PAO1 wild type and Δ*wspF*, PAO1/p*_lac_*-*yhjH*, Δ*wspF* Δ*pscJ*, and Δ*wspF* Δ*pscJ*COM mutants, as described above. At 2 dpi and 7 dpi, the surface of the infected mouse corneal was photographed using slit lamp microscopy (NS-2D; Righton, Tokyo, Japan) under normal light conditions prior to being subjected to dissection for confocal imaging. The dissected corneas were stained for 15 min with 1 μM LysoTracker Red DND-99 (Molecular Probes, USA) and 0.165 μM Alexa Fluor 635-phalloidin (Life Technologies) (633-nm excitation, 647-nm emission), which stain the acidic compartment and F-actin of eukaryotic cells, respectively, before imaging via CLSM.

To determine the bacterial loads, each dissected cornea was placed in a 1.5-mL Eppendorf tube containing 500 μL of sterile 0.9% NaCl and 15 3-mm glass beads (Sigma-Aldrich, USA) before being subjected to vigorous vortexing for 5 min. The suspensions were then serially diluted and plated on LB agar for bacterial enumeration. Mean values and standard deviations (SD) from triplicate experiments are shown.

For cytokine quantification, dissected corneas were placed in a 1.5-mL Eppendorf tube containing 500 μL of sterile 0.9% NaCl before the corneas were crushed into small fragments using a 1.5-mL micropestle (Sigma-Aldrich, USA). The corneas were further homogenized via a VCX 750 Vibra-Cell ultrasonic processor (Sonics & Materials, USA) to lyse the corneal cells. The cell debris was removed via centrifugation (13,000 × *g*, 5 min), and the supernatants were used to characterize the innate immune responses. The cytokine levels were normalized to the total proteins using a Qubit 2.0 fluorometer (Invitrogen, USA) prior to characterization of the proinflammatory response using the Bio-Plex Pro mouse cytokine 8-plex assay (Bio-Rad) with the Bio-Plex 200 system (Bio-Rad, USA). TNF-ɑ concentrations were quantified using the mouse TNF-ɑ enzyme-linked immunosorbent assay (ELISA) Ready-SET-Go! kit (EBioscience, USA) according to the manufacturer’s instructions, and absorbance at 370 nm was measured with Tecan microplate reader Infinite 200 PRO (Tecan, Switzerland). Mean values and SD from triplicate experiments are shown.

### Confocal laser scanning microscopy.

Dissected corneas were visualized under CLSM with either a ×40 or ×63 lens objective. Images were acquired using an argon laser at 488-nm excitation and 535-nm emission for GFP observation and a helium laser at 633-nm excitation and 680-nm emission for STYO 62 or 633-nm excitation and 647-nm emission for Alexa Fluor 635-phalloidin.

### Transcriptomic analysis.

**(i) RNA preparation.** Scratched corneas were infected with the P. aeruginosa PAO1 wild-type or Δ*wspF* or PAO1/p*_lac_*-*yhjH* strain as described above. At the 2- and 7-dpi time points, the infected corneas were dissected and placed in the 1.5-mL Eppendorf tube individually before being dipped into liquid nitrogen to freeze the tissue samples. The frozen tissue samples were crushed into smaller fragments using 1.5-mL size RNase-free micropestle (Sigma-Aldrich, USA) before subjected to total RNA extraction using MiRNeasy minikit (Qiagen, Netherlands). A vigorous Turbo DNA-free protocol was used for DNase treatment (Ambion, USA). The RNA integrity and DNA contamination level were assessed with Agilent 2200 Tapestation (Agilent Technologies, USA) and Qubit 2.0 fluorometer, respectively. Three biological replicates were used for the transcriptomic analysis at each time point.

**(ii) RNA sequencing and data analysis.** Gene expression analysis was conducted via Illumina RNA sequencing (RNA-Seq technology). RNA-Seq was conducted for three biological replicates of each sample. Libraries were produced using an Illumina TruSeq stranded mRNA sample prep kit. The libraries were sequenced using the Illumina HiSeq 2500 platform (Illumina, USA) with a paired-end protocol and read lengths of 100 nucleotides.

Approximately 20 million reads were obtained for each sample. Raw reads were trimmed, adaptor sequences removed, and any reads below 50 bp discarded. Trimmed reads were then mapped onto the mouse reference genome, which can be downloaded from the Ensemble database (ftp.ensembl.org) using the “RNA-Seq and expression analysis” application of the CLC Genomics Workbench 9.0 (CLC Bio, Aarhus, Denmark). The following criteria were used to filter the unique sequence reads: maximum number of hits for a read of 1, minimum length fraction of 0.9, minimum similarity fraction of 0.8 and maximum number of mismatches of 2. A constant of 1 was added to the raw transcript count value to avoid any problems caused by 0.

The raw count table of transcripts was used as an input for the Deseq2 R package for differential expression analysis (72). First, the raw counts were normalized according to the sample library size. Next, a negative binomial test was performed to identify the differentially expressed genes. The transcripts were determined as differentially expressed among pairwise comparisons when their absolute fold change value was greater than 2 and the associated adjusted *P* value was smaller than 0.05. The normalized transcripts were then log_2_(*N* + 1) transformed prior to principal-component analysis (PCA).

**(iii) Functional enrichment of mouse transcriptome.** The functional enrichment of biological process of mouse transcriptomics data was processed using WebGestalt (73, 74). Briefly, the mouse transcriptomics data were uploaded with their gene symbols and respective fold changes as the score. Mus musculus (house mouse) was selected as the reference organism of interest. The uploaded genes were undergone Gene Set Enrichment Analysis (GSEA) using the KEGG pathway as the functional database.

### qRT-PCR analysis.

Total RNA was extracted using an miRNeasy minikit with on-column DNase digestion. The purity and concentration of the RNA were determined by NanoDrop 2000 spectrophotometry (Thermo Fisher Scientific, USA) and the integrity of the RNA was measured using an Agilent 2200 TapeStation system. The contaminating DNA was eliminated using a Turbo DNA-free kit and confirmed by real-time PCR amplification of the *rpoD* gene using total RNA as the template.

Quantitative reverse transcriptase PCR (qRT-PCR) was performed using a two-step method. First-strand cDNA was synthesized from total RNA using the SuperScript III First-Strand Synthesis SuperMix kit (catalog no. 18080-400; Invitrogen). The cDNA was used as a template for qRT-PCR with a SYBR Select Master Mix kit (catalog no. 4472953; Applied Biosystems by Life Technologies) on an Applied Biosystems StepOnePlus real-time PCR system. The *rpoD*, *proC*, and GAPDH (glyceraldehyde-3-phosphate dehydrogenase) genes were used as endogenous controls. All pairs of primers were confirmed to have an efficiency between 90 and 110% before performing the qRT-PCR. Melting curve analyses were employed to verify the specific single-product amplification.

### Data availability.

The dual-species RNA-sequencing data have been deposited in the National Center for Biotechnology Information (BioProject no. PRJNA329171). The RNA-Seq data for P. aeruginosa *in vitro* biofilm cells and planktonic cells were previously published (30) and deposited in the National Center for Biotechnology Information Sequence Read Archive (SRA) database under accession no. SRP041868.
