# Supplementary material for: Elevated c-di-GMP Levels and Expression of the Type III Secretion System Promote Corneal Infection by Pseudomonas aeruginosa
Source: Infect Immun. 2022 Aug 1;90(8):e00061-22. doi: 10.1128/iai.00061-22 (PMC9387266; doi:10.1128/iai.00061-22)
Supplement: Supplemental file 8 — Supplemental material. Download iai.00061-22-s0001.pdf, PDF file, 0.7 MB [file iai.00061-22-s0001.pdf]

Elevated cyclic-di-GMP levels and expression of the type III secretion system promote corneal infection by *Pseudomonas aeruginosa*

Joey Kuok Hoong Yam [1]Ɣ, Thet Tun Aung [2,3] Ɣ, Song Lin Chua [4], Yingying Cheng [5], Gurjeet Singh Kohli [6], Jianuan Zhou [7], Florentin Constancias [8], Yang Liu [9], Zhao Cai [9], May Margarette Santillan Salido [1], Daniela I. Drautz-Moses [1], Scott A. Rice [1,10,11], Stephan Christoph Schuster [1,10], Zhao Zhi Boo [10], Bin Wu [10], Staffan Kjelleberg [1,10,12], Tim Tolker-Nielsen [13], Rajamani Lakshminarayanan [2,14,15], Roger W. Beuerman [2,16,17,18], Liang Yang [9*], Michael Givskov [1,13*]

Table S1. Strains and primers used in this study.

Strains and primers	Relevant characteristic(s)	Source or reference
Strains		
PAO1	Prototypic wild-type strain	1
Δ*wspF*	*wspF* derivative of PAO1 constructed by allelic exchange	2
Δ*siaD*	*siaD* derivative of PAO1 constructed by allelic exchange	3
Δ*PA5442*	*PA5442* derivative of PAO1 constructed by allelic exchange	This study
Δ*wspF*Δ*pscJ*	*pscJ* derivative of Δ*wspF* constructed by allelic exchange	This study
Δ*wspF*Δ*pscJ*COM	Δ*wspF*Δ*pscJ* mutant complemented by wild-type *pscJ* gene via the miniCTX2-*pscJ*	This study
PAO1/p_{lac}-*yhjH*	Tc^r; PAO1 containing the p_{lac}-*yhjH* vector	4
PAO1/p_{cdrA}-*gfp*	Gm^r; PAO1 carrying the p_{cdrA}-*gfp* report	5
PAO1-miniTn7-p_{lac}-*gfp*	Gm^r; PAO1 tagged by the miniTn7-gfp	4
Δ*wspF*-miniTn7-p_{lac}-*gfp*	Gm^r; Δ*wspF* tagged by the miniTn7-gfp	4

PAO1/p_{lac}-*yhjH* - miniTn7- p_{lac}-*gfp*	Gm^r; Tc^r; PAO1/p_{lac}-*yhjH* tagged by the miniTn7-gfp	[4]
PAO1/p_{exoT}-*gfp*	Gm^r; PAO1 carrying the miniTn7-*exoT-gfp* report	[6]
Primers for constructing the deletion mutant		
PA5442-1	agctcggtacccgggTGAACTGCTGGATGTTCTCG	
PA5442-2	TTCAACGAGCCTGGCGCAAAGTCATAGGCGCTTGTTCTTG	
PA5442-3	CAAGAACAAGCGCCTATGACTTTGCGCCAGGCTCGTTGAA	
PA5442-4	cgacggccagtgccaATCTTTTCCGGTAGCGCTTC	
PA5442-F	AACGGTAGTTCTGCTCCAG	
PA5442-R	CTCGCCCTCGAAGAATTCG	
pscJ-1	agctcggtacccgggACATCAACTCGACGGTGACC	
pscJ-2	AGAAGAACTGCGCCAGGTTGACAGACCTTTCACCGTTCGC	
pscJ-3	GCGAACGGTGAAAGGTCTGTCAACCTGGCGCAGTTCTTCT	
pscJ-4	cgacggccagtgccaGCATTCATCGCTCGCTTGTC	
pscJ-F	CCGAGCTGCAACACAAGATC	
pscJ-R	GCGACTGGCGTCTAGTTCAA	
pscJCOM-F	CGGGATCCCGAGATCAGGGAGCGTTTCAGC	
pscJCOM-R	GGAATTCCCGCAGTCCAATTCCAGTTGC	
Primers for qRT-PCR analysis		
pelA-F	GACAACTGGATCGCTGCACG	
pelA-R	CTTGAGTTTCTGCGACAGCC	
cupA1-F	TATCACATTCAGCGGCGAAG	
cupA1-R	TCGGTGAGTTGCAGGGTGAAG	
dipA-F	AAGAACCACGAGCCTTACTG	
dipA–R	CCAGCTTGTTCTGCGTGATG	
nbdA-F	CGGCATTCAACGAAGTGTTC	
nbdA-R	TCGTTGATCCGCTTGAAGTG	
PA0575-F	CCGAGTACGCCATCCACTAC	

PA0575-R	TGATCGGGGTGACCAAATCG
PA1181-F	TCAACAGCGACATCACCG
PA1181-R	CCGATGGAATACAGGGTCAC
PA2072-F	GGCATTTCGTCTTCACCTATC
PA2072-R	CCTTCCAGCCACTTGACGAT
mucR-F	TCAGCCAGCCGTACCAGATA
mucR-R	CTGGTCCTTGGCGTGATACA
GAPDH-F	GACCGTTCCTCGACAAGTTC
GAPDH-R	TCAGCAGGCCTTCGAGATAG
proC-F	ATCAGTTGCTCCGGCTTCAG
proC-R	ATCGACGTGGTCGAGTCCAA
rpoD-F	GGGGCTGTCTCGAATACGTT
rpoD-R	GGGATACCTGACTTACGCGG

Table S2. Mapping statistics of the RNA sequencing raw reads of 2 dpi

	**PAO1_2dpi_R1**	**PAO1_2dpi_R2**	**PAO1_2dpi_R3**	***ΔwspF*_2dpi_R1**	***ΔwspF*_2dpi_R2**	***ΔwspF*_2dpi_R3**	**yhjH_2dpi_R1**	***yhjH*_2dpi_R2**	***yhjH*_2dpi_R3**
**Total reads obtained**	40,035,814	42,736,300	45,704,980	38,877,460	43,753,686	44,780,662	44,881,008	44,024,644	39,339,728
**Total reads after trimming**	38,346,440	40,895,410	44,112,144	37,145,526	41,698,876	42,801,172	43,227,742	42,296,266	37,700,828
**Total reads mapped in pairs to *P. aeruginosa***	1,175,098	716,482	547,026	1,192,838	1,092,994	1,018,384	1,088,030	631,282	404,204
**Total reads mapped in pairs to *P. aeruginosa* (%)**	3.06	1.75	1.24	3.21	2.62	2.38	2.52	1.49	1.07
**Total fragments mapped to *P. aeruginosa***	587,549	358,241	273,398	596,419	546,497	509,192	544,015	315,641	201,774
**Fragments mapped to *P. aeruginosa* coding genes**	513,777 (87.44%)	312,588 (87.26%)	236,872 (86.60%)	516,942 (86.67%)	472,474 (86.46%)	448,172 (88.02%)	474,418 (87.21%)	276,644 (87.65%)	177,663 (87.91%)
**Fragments mapped to *P. aeruginosa* intergenic regions**	73,772 (12.56%)	45,653 (12.74%)	36,641 (13.40%)	79,477 (13.33%)	74,023 (13.54%)	61,020 (11.98%)	69,597 (12.79%)	38,997 (12.35%)	24,439 (12.09%)
**Total reads mapped in pairs to mouse**	26,460,142	29,463,476	31,741,164	25,457,626	28,428,862	29,433,704	30,113,868	30,435,404	26,269,540
**Total reads mapped in pairs to mouse (%)**	69.00	72.05	71.96	68.53	68.18	68.77	69.66	71.96	69.68

Table S3. Mapping statistics of the RNA sequencing raw reads of 7 dpi

	**PAO1_7dpi_R1**	**PAO1_7dpi_R2**	**PAO1_7dpi_R3**	***ΔwspF*_7dpi_R1**	***ΔwspF*_7dpi_R2**	***ΔwspF*_7dpi_R3**	**yhjH_7dpi_R1**	***yhjH*_7dpi_R2**	***yhjH*_7dpi_R3**
**Total reads obtained**	36,342,580	33,049,080	40,675,146	41,007,268	42,895,076	42,955,498	40,264,878	41,793,392	37,251,962
**Total reads after trimming**	35,621,656	32,490,358	39,353,788	39,629,118	41,411,038	41,401,708	39,009,538	40,425,854	36,351,936
**Total reads mapped in pairs to *P. aeruginosa***	107,380	36,888	63,184	6,066	7,444	3,238	21,574	39,896	81,036
**Total reads mapped in pairs to *P. aeruginosa* (%)**	0.30	0.11	0.16	0.02	0.02	0.01	0.06	0.10	0.22
**Total fragments mapped to *P. aeruginosa***	53,690	18,444	31,592	3,033	3,722	1,619	10,787	19,948	40,518
**Fragments mapped to *P. aeruginosa* coding genes**	45,974 (85.63%)	16,268 (88.20%)	26,903 (85.16%)	2,635 (86.88%)	3,151 (84.66%)	1,384 (85.48%)	9,302 (86.23%)	17,160 (86.02%)	34,931 (86.21%)
**Fragments mapped to *P. aeruginosa* intergenic regions**	7,716 (14.37%)	2,172 (11.80%)	4,689 (14.84%)	398 (13.12%)	571 (15.34%)	235 (14.52%)	1,485 (13.77%)	2,788 (13.98%)	5,587 (13.79%)
**Total reads mapped in pairs to mouse**	28,087,174	25,627,294	30,082,942	31,136,034	31,910,616	32,332,158	30,199,178	31,233,596	28,989,592
**Total reads mapped in pairs to mouse (%)**	78.85	78.88	76.44	78.57	77.06	78.09	77.41	77.26	79.75

Table S4. Statistics of differentially expressed bacterial genes for each transcriptomic comparison group

**Comparison**	**Differentially expressed genes**				
	**Up-regulation**	**Down-regulation**	**Between 2 to 4-fold change**	**More than 4-fold change**	**Total**
in vivo PAO1 vs *in vitro* planktonic cells	979	1082	1072	989	2061
in vitro biofilm cells vs *in vitro* planktonic cells	1004	1014	1261	757	2018
in vivo Δ*wspF* vs *in vivo* p_{lac}-*yhjH*-containing strain	46	87	112	21	133
in vivo PAO1 vs *in vivo* p_{lac}-*yhjH*-containing strain	49	79	112	16	128
in vivo PAO1 vs *in vitro* biofilm cells	900	897	977	820	1797

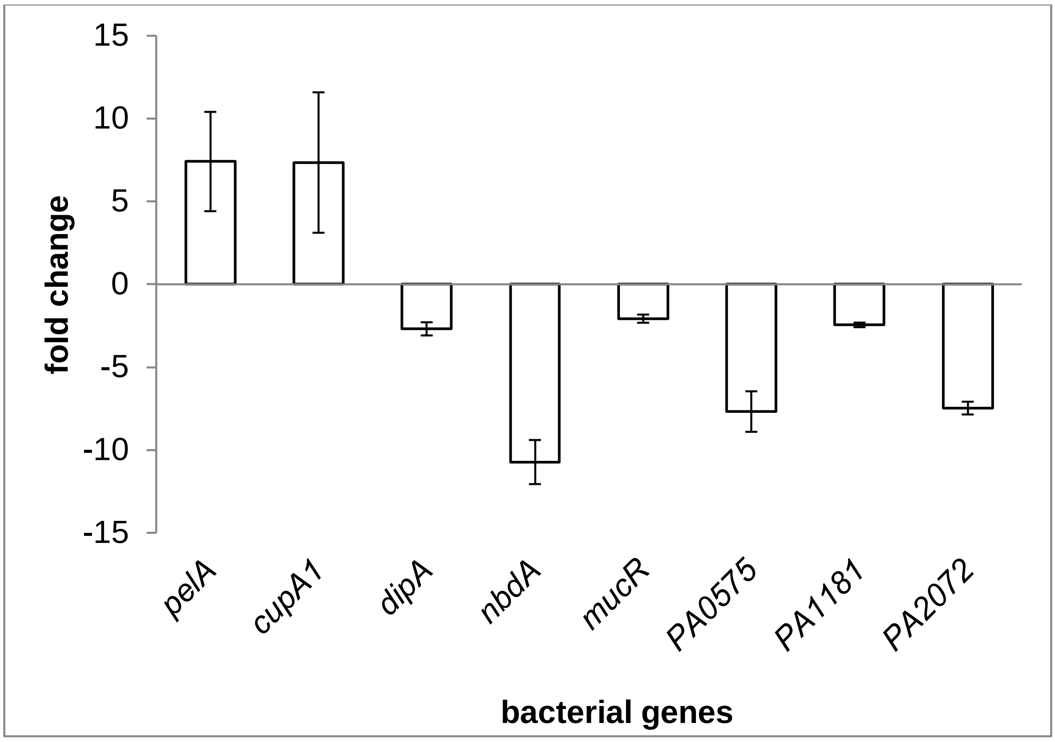

Figure S1. qRT-PCR analysis. Expression of *pelA*, *cupA1*, *dipA*, *mucR*, *nbdA*, *PA0575*, *PA1181*, *PA2072* messenger RNA in 2 dpi *in vivo* PAO1 compared with *in vitro* planktonic cells. Means and s.d. from triplicate experiments are shown. *p < 0.01.

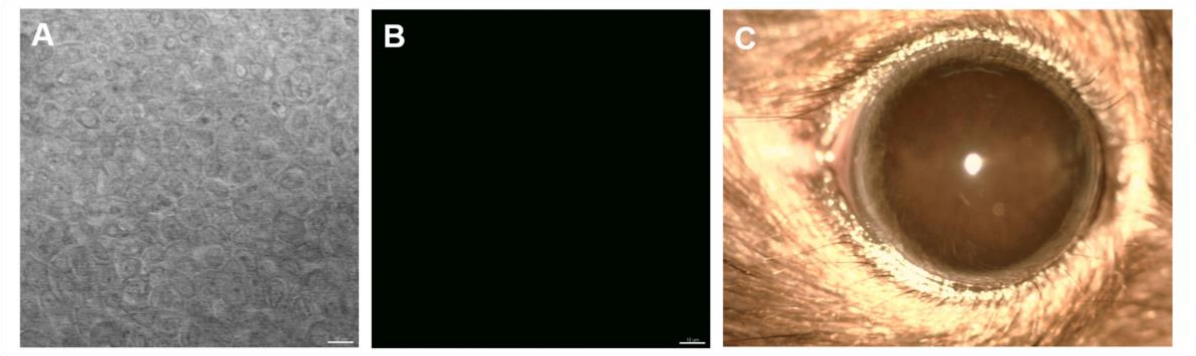

Figure S2. Images of control uninfected mouse corneas. The uninfected mouse corneas were viewed under bright field (A), CLSM (green fluorescence channel) (B) and slit lamp microscopy (C). Experiments were performed in triplicate, and a representative image for each condition is shown. Scale bars, 10 μm.

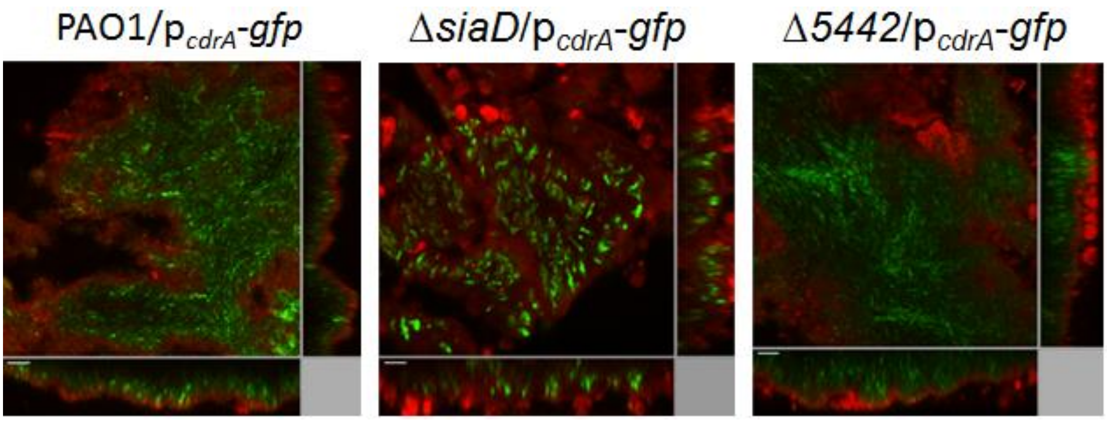

Figure S3. p$_{cdrA}$-*gfp* expression in different *P. aeruginosa* DGC mutants (derived from PAO1 wild type) during 2 dpi ocular infections. Experiments were performed in triplicate, and a representative image for each condition is shown. Green and red fluorescence refer to the expression of p$_{cdrA}$-*gfp* expression and SYTO®62 stain respectively. Scale bars, 20 μm.

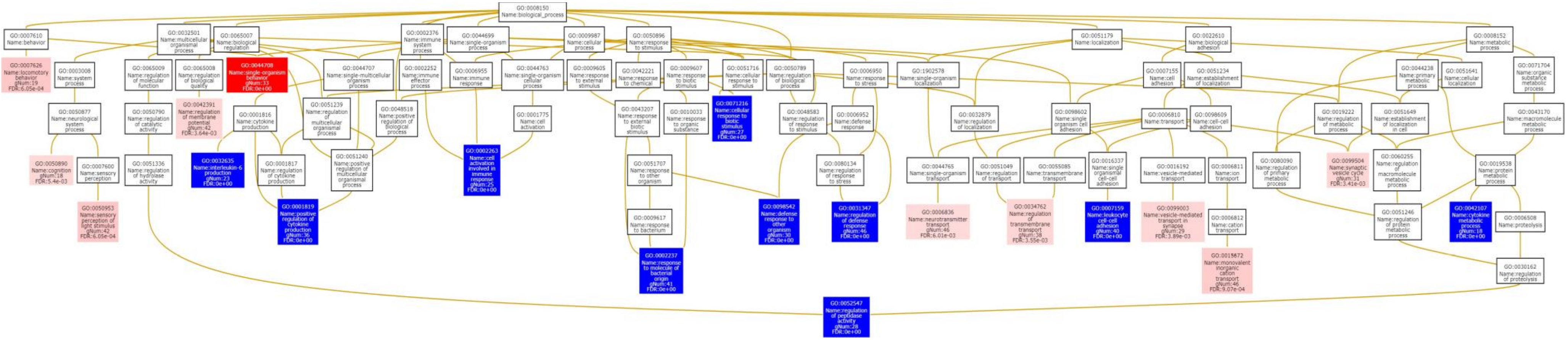

Figure S4. Biological process enrichment of mouse transcriptome using WEB-based GEne SeT AnaLysis Toolkit (WebGestalt) by comparing 7 dpi to 2 dpi Δ*wspF*-infected corneal cells RNA-seq data from supplementary data 6. The grouping and classification of each biological process was processed by WebGestalt. The groups highlighted in pink or red indicate the weak or strong upregulation of gene expression in 7 dpi comparing to 2 dpi respectively. The groups highlighted in light blue or dark blue refers to weak or strong downregulation of gene expression in 7 dpi than 2 dpi respectively.

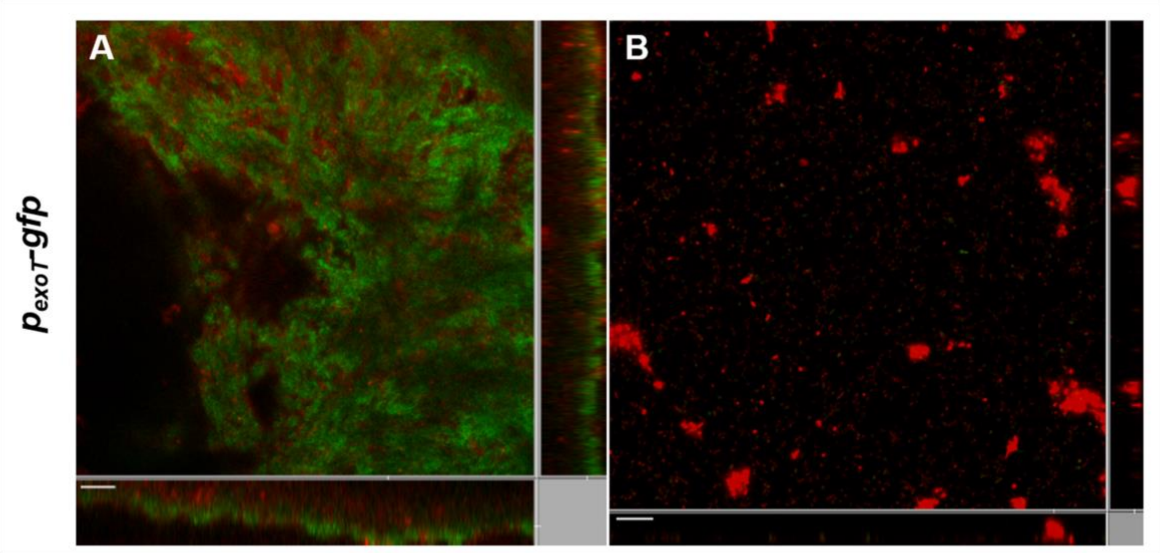

Figure S5. Confocal images of *P. aeruginosa* the T3SS reporter strain, p_{exoT}*-gfp*, after cultivation *in vivo* and *in vitro* for 2 days. A) Mouse cornea after 2 days infection with the p_{exoT}-*gfp* strain. B) 2 days old *in vitro* static biofilm formed by the p_{exoT}-*gfp* strain. Green and red fluorescence represents the expression of p_{exoT}-*gfp* and staining by SYTO®62. Experiments were carried out in triplicates and a representative image is shown for each condition. Scale bars, 20 μm.

Supplementary data 1. Regulated genes in 2 dpi *in vivo* PAO1 compared to *in vitro* planktonic cells.

Supplementary data 2. Regulated genes in *in vitro* biofilm cells compared to *in vitro* planktonic cells.

Supplementary data 3. Shared regulated genes between 2 dpi *in vivo* PAO1 and *in vitro biofilm* cells when compared to *in vitro* planktonic cells.

Supplementary data 4. Regulated genes in 2 dpi *in vivo* Δ*wspF* compared to 2 dpi *in vivo* p_{lac}-*yhjH*-containing strain.

Supplementary data 5. Regulated genes 2 dpi *in vivo* PAO1 compared to 2 dpi *in vivo* p_{lac}-*yhjH*-containing strain

Supplementary data 6. Regulated genes in 7 dpi corneal cells compared to 2 dpi corneal cells after infected by Δ*wspF* strain.

Supplementary data 7. Regulated genes in 2 dpi *in vivo* PAO1 compared to *in vitro* biofilm cells.

References

1. Holloway, B.W. & Morgan, A.F. Genome organization in Pseudomonas. *Annu Rev Microbiol* **40**, 79-105 (1986).

2. Rybtke, M.T. *et al.* Fluorescence-based reporter for gauging cyclic di-GMP levels in Pseudomonas aeruginosa. *Appl Environ Microbiol* **78**, 5060-5069 (2012).

3. Chua, S.L. *et al.* C-di-GMP regulates Pseudomonas aeruginosa stress response to tellurite during both planktonic and biofilm modes of growth. *Scientific reports* **5**, 10052 (2015).

4. Chua, S.L. *et al.* Bis-(3'-5')-cyclic dimeric GMP regulates antimicrobial peptide resistance in Pseudomonas aeruginosa. *Antimicrob Agents Chemother* **57**, 2066-2075 (2013).

5. Wolcott, R.D. & Ehrlich, G.D. Biofilms and chronic infections. *JAMA* **299**, 2682-2684 (2008).

6. Almblad, H. *et al.* The Cyclic AMP-Vfr Signaling Pathway in Pseudomonas aeruginosa Is Inhibited by Cyclic Di-GMP. *J Bacteriol* **197**, 2190-2200 (2015).
